# Avirulent Isolates of *Penicillium chrysogenum* to Control the Blue Mold of Apple Caused by *P. expansum*

**DOI:** 10.3390/microorganisms11112792

**Published:** 2023-11-17

**Authors:** Holly P. Bartholomew, Dianiris Luciano-Rosario, Michael J. Bradshaw, Verneta L. Gaskins, Hui Peng, Jorge M. Fonseca, Wayne M. Jurick

**Affiliations:** 1 Food Quality Laboratory, Beltsville Agricultural Research Center, Agricultural Research Service, United States Department of Agriculture, Beltsville, MD 20705, USA; 2 Department of Organismic & Evolutionary Biology, Harvard University, Cambridge, MA 02138, USA

**Keywords:** apple fruit, biocontrol, blue mold, mycotoxin, patulin, *Penicillium chrysogenum*, *Penicillium expansum*, postharvest decay

## Abstract

Blue mold is an economically significant postharvest disease of pome fruit that is primarily caused by *Penicillium expansum*. To manage this disease and sustain product quality, novel decay intervention strategies are needed that also maintain long-term efficacy. Biocontrol organisms and natural products are promising tools for managing postharvest diseases. Here, two *Penicillium chrysogenum* isolates, 404 and 413, were investigated as potential biocontrol agents against *P. expansum* in apple. Notably, 404 and 413 were non-pathogenic in apple, yet they grew vigorously in vitro when compared to the highly aggressive *P. expansum* R19 and Pe21 isolates. Whole-genome sequencing and species-specific barcoding identified both strains as *P. chrysogenum*. Each *P. chrysogenum* strain was inoculated in apple with the subsequent co-inoculation of R19 or Pe21 simultaneously, 3, or 7 days after prior inoculation with 404 or 413. The co-inoculation of these isolates showed reduced decay incidence and severity, with the most significant reduction from the longer establishment of *P. chrysogenum*. In vitro growth showed no antagonism between species, further suggesting competitive niche colonization as the mode of action for decay reduction. Both *P. chrysogenum* isolates had incomplete patulin gene clusters but tolerated patulin treatment. Finally, hygromycin resistance was observed for both *P. chrysogenum* isolates, yet they are not multiresistant to apple postharvest fungicides. Overall, we demonstrate the translative potential of *P. chrysogenum* to serve as an effective biocontrol agent against blue mold decay in apples, pending practical optimization and formulation.

## 1. Introduction

Postharvest diseases are an economically devastating global problem and result in major losses annually [1]. One of the most prevalent postharvest diseases is blue mold, caused by *Penicillium* spp., which targets pome fruit during short- and long-term storage (e.g., apple, pear, quince) [2]. Symptoms of blue mold decay appear after wounded fruits are colonized by the fungus, where they proliferate and cause a soft, watery brown lesion, with a well-defined margin. Besides rot, another major form of postharvest loss is from mycotoxins produced by these fungi [3]. Patulin, a mycotoxin produced by *Penicillium expansum*, is one of the most problematic due to the harmful effects it causes in humans, including cytotoxic, genotoxic, and acute forms of damage [4,5,6]. Given the stability of the molecule during pasteurization in conjunction with the human health impacts, regulations are in place at a global scale to reduce instances of patulin contamination in processed pome fruit products [7,8].

Due to the problematic nature of food loss, waste, and toxin contamination, there are many efforts to mitigate blue mold decay in fruit and processed fruit products. When harvested, apples are washed and sorted based on size and quality, and then treated with postharvest fungicides via bin drenches, dips, line sprays, and/or thermofogged [9,10]. Four fungicides are approved for apple postharvest use in the United States: thiabendazole (FRAC 1), pyrimethanil (FRAC 9), fludioxonil (FRAC 12), and difenoconazole (FRAC 3) [9]. However, resistance in *Penicillium* spp. to each of these different fungicides has been detected, including instances of multidrug resistance [11,12,13,14,15,16]. To combat fungicide resistance, rotation (based on FRAC code) is combined with fungicide mixes comprising multiple mode-of-action chemistries (e.g., Academy (Syngenta Crop Protection, Greensboro, NC, USA), which contains fludioxonil and difenoconazole). Even so, with the rise in single, double, and multidrug-resistant isolates, further investigation into resistance mechanisms, the discovery of new mode-of-action materials, and alternative strategies that are appropriate for conventional and organic production are critical to maintaining fruit quality during storage.

One alternative approach to reduce fungal rot and concomitant mycotoxin production is via the use of biocontrol organisms. Biocontrols are live organisms that are used to combat the colonization or proliferation of pathogenic microbes. Biocontrol strategies rely on different mechanisms like (1) niche competition between the antagonistic organism and pathogen; (2) direct targeting or the inhibition of the unwanted phytopathogen; (3) influencing the plant or produce so as to induce its own resistance; or (4) through the production of fungistatic or fungicidal secondary metabolites or volatile organic compounds (VOCs) [3,17,18]. Afla-guard^®^ GR (Syngenta, Basel, Switzerland), an atoxigenic isolate of *Aspergillus flavus*, has been implemented in the field for corn and peanut products to protect against *A. flavus* aflatoxin-producing strains via direct competition [19]. In a postharvest context, there have been extensive efforts to find antagonistic organisms against blue mold in apple, such as the yeast *Hannaella sinensis* [20]. There is a commercial biocontrol product approved by the Food and Drug Administration (FDA) for blue mold decay in the USA, called Bio-Save^®^ (Jet Harvest Solutions, Longwood, FL, USA). Bio-Save^®^ contains the bacterium *Pseudomonas syringae* and works by coating the fruit prior to storage for direct niche competition with blue mold organisms in storage for pome fruit and sweet potato [21,22]. However, with a drastic rise in fungicide resistance and the need for protection against a broad range of pathogens, the development of multiple strategies to combat these postharvest fungal diseases is critical.

To determine additional organisms for use as a biocontrol, investigation into their toxic potential and pathogenicity in vivo is necessary. Therefore, the goal of this study was to investigate the biocontrol potential of two isolates to reduce blue mold decay incidence and severity. In this study, we discovered two independent and avirulent (in apple) *Penicillium chrysogenum* isolates and compared them at the biological, genomic, and mycotoxigenic levels with two highly aggressive *P. expansum* strains. Furthermore, we provide in vivo evidence that the described strains can outcompete pathogenic blue mold strains when applied with adequate time for the colonization of *P. chrysogenum*. Ultimately, this study lends a solid foundation for their optimization and eventual potential as an alternative to chemical fungicides.

## 2. Materials and Methods

### 2.1. Fungal Isolate Propagation, Media, and Storage

*Penicillium chrysogenum* 404 and *P. chrysogenum* 413 (hereby referred to as 404 and 413) were initially obtained from a hygromycin-B-amended PDA medium (100 ppm) colonized by airborne conidia in the laboratory, and the single spore isolation method was performed as previously described [23]. *Penicillium expansum* isolates R19 and Pe21 (now R19 and Pe21) were utilized from the lab collection [24,25]. Three *Penicillium rubens* (DTO 346-D4, DTO 397-I3, and DTO 405-A6) and four *P. chrysogenum* (DTO 275-D7, DTO 382-H5, DTO 402-A9, and DTO 431-E3) type specimens were obtained from the Westerdjik Institute in the Netherlands. All fungal isolates (Table 1) were maintained in long-term glycerol storage at −80 °C and propagated on potato dextrose agar (PDA).

For DNA sequencing, 404 and 413 were grown on PDA plates for 7 days at 25 °C. Conidia were harvested (100 µL of 1 × 10^6^ conidia/mL in dH_2_O) and used to inoculate 50 mL of PDB. Liquid shake cultures were grown for 7 days in a temperature-controlled incubator at 23 °C and 150 rpm. Cultures were individually vacuum-filtered with a 0.2 µM cellulose acetate filter, and the mycelial mat, needed for DNA extractions, was aseptically removed from the filter and placed in a 50 mL conical tube. All tubes were immediately submerged in liquid N_2_ and then stored at −80 °C.

### 2.2. Virulence Assays in Apple Fruit

‘Gala’ (MOM’s Organic Market), ‘Golden Delicious’ (Penn State Fruit Research and Extension Center, Biglerville, PA, USA), ‘Honeycrisp’ (MOM’s Organic Market), and ‘Fuji’ (MOM’s Organic Market) apples were obtained for experiments. To estimate the physiological maturity of the fruit, five apples of each cultivar were evaluated with a starch-iodine (I2KI) test and compared to the Cornell Starch Index Scale as previously described [26]. All apples used for subsequent assays exhibited late-stage maturity (7–8) on the scale. Apple inoculations were performed as described for two independent trials [23,27]. The fungal conidial suspensions of R19, Pe21, 404, and 413 were adjusted to 1 × 10^5^ conidia/mL, and 10 µL was used to inoculate into each wound, totaling 5 apples of 4 cultivars (‘Fuji’, ‘Gala’, ‘Golden Delicious’, and ‘Honeycrisp’) per strain, including a TTW control. Apples were placed on packing trays and stored in standard apple-packing boxes at room temperature (22–25 °C) for 7 days. Photographs of the wound site and measurements of lesion diameters were recorded for each apple at 3, 5, and 7 days postinoculation. This assay was executed twice.

To confirm the viability of each inoculated strain, apple tissue around the initial wound area (404, 413, and TTW) or the leading edge of the lesion (R19 and Pe21) on day 7 postinoculation was aseptically transferred with a sterile scalpel to the PDA and PDA amended with hygromycin (100 ppm) for each isolate. Plates were incubated for 7 days at 25 °C and examined for fungal growth on the agar. This was performed in duplicate for each of the two trials.

To test the virulence of the well-characterized type specimens of *P. chrysogenum* and *P. rubens* (listed in Table 1), 5 apples of cultivars ‘Golden Delicious’, ‘Honeycrisp’, and ‘Fuji’ were inoculated as above for each type specimen isolate, and for 404, 413, R19, and TTW control for comparison. These fruits were stored at room temperature for 14 days before the measurement and assessment of lesion formation. For each type specimen, strain viability from inoculated apple tissue was repeated with isolation on the PDA medium. Apple inoculation and the re-isolation of the strains from inoculated apple fruits were performed twice.

For competitive evaluation in apple fruit between the two fungal species, 5 ‘Honeycrisp’ apples/inoculum were washed and wounded as previously described. Fungal strains R19, Pe21, 404, and 413 were grown on the PDA for 7 days at 25 °C, and conidia were harvested as described above. The 404 and 413 fungal conidial suspensions were adjusted to 1 × 10^7^ conidia/mL, and 10 µL was used to inoculate into each wound, alongside the TTW control. For co-inoculation, R19 and Pe21 conidial suspensions were adjusted to 1 × 10^5^ conidia/mL, and 10 µL was added to each wound 1 h (0 days), 3 days, or 7 days after the inoculation of the initial 404, 413, or TTW. All conidial suspensions were harvested on the same day as inoculation. Apples were stored at room temperature following the initial inoculation and through the duration of the experiment. Lesion diameter measurements and photographs of each apple were taken 3, 5, and 7 days after the inoculation of the second isolate (e.g., either Pe21 or R19) for two trials.

To evaluate the cultivar impact on the competition between isolates, the above experiment was repeated with ‘Gala’ apples using only the 7-day inoculation delay between initial and secondary inoculation.

### 2.3. Fungal Germination and Mycelial Growth Assessment

Germination and radial growth assays were performed as previously described [26,27]. Briefly, conidia were harvested for R19, Pe21, 404, and 413 and adjusted to 1 × 10^5^ conidia/mL in sterile dH_2_O, and 10 µL was inoculated onto the center of PDA plates. Six plates per isolate (three for germination assay and three for radial growth) were wrapped in parafilm and incubated at 25 °C. Germination percentage was assessed 14 and 24 h postinoculation. The diameter of each colony was measured 3, 5, and 7 days postinoculation. Each experiment was conducted two times.

To evaluate in vitro antagonism between isolates, R19, Pe21, 404, and 413 conidia were harvested and adjusted to 1 × 10^5^ conidia/mL. The 404 and 413 isolates were inoculated onto the center of a 100 mm × 15 mm PDA plate (10 µL conidial suspension) three times, and R19 and Pe21 isolates were inoculated 1 h (0 days), 3 days, or 7 days after the initial inoculum, approximately 10-20 mm to either side of the initial inoculation area or leading edge of the adjacent colony. As a control, the 404 and 413 isolates were inoculated on the plate opposite the side of R19 and Pe21. Plates were photographed and observed at 3, 5, and 7 days after the inoculation of the second isolate. This experiment was performed twice.

### 2.4. Evaluation of Antimicrobial Resistance

To assess antimicrobial resistance, isolates were grown using the following fungicide-amended media: PDA only, hygromycin B (100 ppm; PDA + HYG), pyrimethanil (0.5 ppm; Sugar Agar + PYR), fludioxonil (0.5 ppm; PDA + FLU), thiabendazole (10 ppm; PDA + TBZ), and difenoconazole (2.5 ppm; Malt Extract Agar + DIF). Conidia were harvested for R19, Pe21, 404, and 413 as described in the growth assay section above. Each strain was used to inoculate the plates three times (10 µL of conidial suspension/inoculation point), incubated at 25 °C for 7 days, and evaluated qualitatively for growth. In addition to the above fungicides, tolerance to the mycotoxin patulin was also investigated as previously described [26,28], using a final patulin concentration of 138 µg/spore suspension or water. Conidial germination was determined at 14 h and 24 h, and the colony diameter of each strain was measured after 7 days.

To assess hygromycin resistance across the *P. chrysogenum* and *P. rubens* isolates, all seven type specimens were plated as described above onto PDA + HYG. Mycelial mat formation was determined qualitatively after 7 days of incubation at 25 °C.

### 2.5. Fungal DNA Extraction and Sequencing

To extract DNA, mycelial mats of 404 and 413 were first ground using a sterile mortar and pestle with liquid N_2_ to a fine powder. DNA was extracted with a Wizard DNA Extraction Kit (Promega, Madison, WI, USA) using the manufacturer’s protocol outlined in the “Isolating Genomic DNA from Plant Tissue” portion of the manual. DNA was rehydrated with nuclease-free sterile water at 4 °C. Samples were then extracted with a phenol–chloroform extraction method per the manufacturer’s protocol (Pacific Biosciences, Menlo Park, CA, USA) using nuclease-free sterile water in place of elution buffer. Sample concentration and quality were determined with a Nanodrop (Thermo Scientific, Waltham, MA, USA) and gel electrophoresis (0.8% agarose in 1X TBE (Bio-Rad Laboratories, Hercules, CA, USA), 90 volts for 45 min), using uncut lambda DNA dilution series as a standard. High-quality samples were sent to Beijing Genomics Institute (BGI) for quality control using a Qubit fluorometer (Thermo Fisher Scientific, Waltham, MA, USA) and 250 bp paired-end sequencing with a DNB-Seq platform (MGI, Shenzhen, China).

### 2.6. Fungal Genome Assembly and Characterization

Data retrieved from BGI were imported into Geneious^®^ (Biomatters, Inc., Boston, MA, USA) with forward and reverse reads compiled into paired-end sets. Sample files were trimmed using BBDuk to a quality filter and trim adapters using default settings. Genome-assisted speciation was performed for 404 and 413 by finding the assembly for each of the annotated genes as benchmark barcodes, namely *BenA*, ITS, *rpb1*, *rpb2*, and *CaM*, and the isolates were then searched against the BLASTn (NCBI, Bethesda, MD, USA) sequence databases to find highly similar sequences (percent identity) across the entire query from type specimens and reference isolates. Both 404 and 413 were assembled using the “Map to Reference” function (Low sensitivity/Fastest parameter with default of 5 iterations) with the annotated *Penicillium rubens* (previously *P. chrysogenum*) P2niaD18 genome (NCBI accession number: CM002798.1) as the reference sequence. To determine patulin gene cluster presence, the known cluster was either used as a reference map for the raw *P. chrysogenum* 404 and 413 sequencing data (also using the above default parameters), or the homologous regions were found within the genome (for R19 and Pe21 assembled genomes) [25,29]. For amino acid similarity, each amino acid sequence in the patulin gene cluster was mapped to *P. chrysogenum* in the NCBI BLASTp database. The highest homology results (percent identity and query cover) were then converted to nucleotides and used as reference maps for the genomic data in Geneious. This was repeated for hygromycin-resistant genes encoding APH(4)-Ia and APH(7″)-Ia.

### 2.7. Statistical Analyses

Statistical analyses were performed using R version 4.20 [30]. A Levene’s test was used to determine the homogeneity of variances because multiple trials were conducted. Data were combined for trials with no significant difference between them (*p* < 0.05). To determine the normality of a dataset, a Shapiro–Wilks test was performed (*p* < 0.05). For data with normal distribution or for those that could satisfy normalcy after transformation, an ANOVA was performed to determine significance, followed by a Tukey’s HSD post hoc test (e.g., mean colony diameter of isolates). If the normal dataset consisted of only two independent groups (patulin and hygromycin germination data for each isolate), a Student’s *t*-test was performed. For non-normal datasets, the non-parametric Kruskal–Wallis test was implemented with a pairwise Wilcox post hoc test with a Benjamin–Hochberg p-adjustment method. For non-normal datasets with two independent groups (e.g., patulin and hygromycin germination data), a Mann–Whitney U test was performed. All error bars in all graphs were generated using the standard deviation.

### 2.8. Phylogenetic Tree

A phylogenetic tree was constructed from loci for the *BenA*, ITS, *rpb1*, *rpb2*, and *CaM* sequences of *P. chrysogenum* and other closely related *Penicillium* species from *Penicillium* sect. *Chrysogena*. Taxa and loci were selected based on the phylogenetic analyses by Houbraken et al. [31]. Sequences were aligned and edited using MUSCLE in MEGA11:Molecular Evolutionary Genetics Analysis, version 11 [32]. A GTR + G + I evolutionary model was used for phylogenetic analyses as it is the most inclusive model of evolution and includes all other evolutionary models [33]. A fixed parameter-rich model (such as GTR + G + I) can be used in lieu of running a test to select the most suitable evolutionary model [33]. The phylogeny was inferred using the Bayesian analysis of the combined *BenA*, ITS, *rpb1*, *rpb2*, and *CaM* using a Yule tree prior [34] and a strict molecular clock, in the program BEAST version 1.10.4 [35]. A single MCMC chain of 10^6^ steps was run, with a burn-in of 10%. Posterior probabilities were calculated from the remaining 9000 sampled trees. A maximum clade credibility tree was produced using TreeAnnotator version 1.10.4 (part of the BEAST package). Stationarity was confirmed by running the analysis multiple times, which revealed convergence between runs. The resulting tree was visualized using FigTree ver. 1.3.1 [36]. A maximum likelihood analysis was performed using raxmlGUI [37] under the default settings with a GTR + G + I evolutionary model. Bootstrap analyses were conducted using 1000 replications [38].

## 3. Results

### 3.1. Isolates 404 and 413 Are Identified as Penicillium chrysogenum

**Figure 1:**
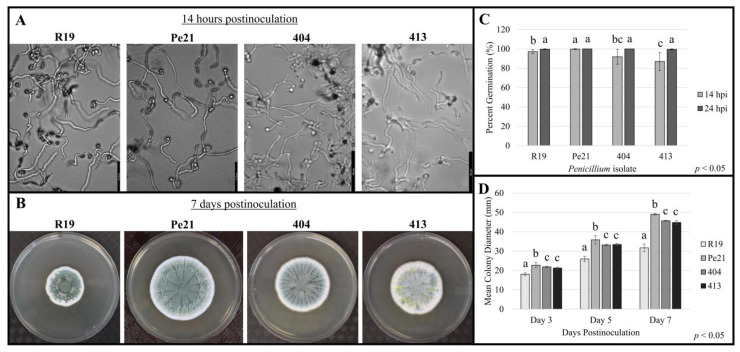
Conidial germination and mycelial growth of *Penicillium chrysogenum* isolates 404 and 413, compared to *P. expansum* strains R19 and Pe21: (**A**) Germinating conidia of R19, Pe21, 404, and 413 on PDA 14 h postinoculation. (**B**) Fungal colonies of R19, Pe21, 404, and 413 on PDA after 7 days. (**C**) Percent conidial germination after 14 and 24 h on PDA. Error bars represent the standard deviation across two independent replicates. A Kruskal–Wallis test was performed to determine significance across isolates for each individual timepoint, followed by a Wilcoxon post hoc test (*p* < 0.05, *n* = 6). (**D**) Mean colony diameter of R19, Pe21, 404, and 413 after 3, 5, and 7 days on PDA. Error bars represent the standard deviation across two independent replicates. An ANOVA test was performed to determine significance across isolates at each timepoint, followed by a Tukey’s HSD post hoc test (*p* < 0.05, *n* = 6) Values with the same letter are not significantly different. Scale bar on microscopy images set to 127.9 µm length for R19 and Pe21, and 64 µm length for 404 and 413.

*Penicillium* spp. isolates 404 and 413 were originally isolated from hygromycin-amended media while culturing hygromycin-resistant *Penicillium expansum* transformants. The colonies of isolates 404 and 413 have a blue-green color with white margins on the PDA medium. Colonies are circular, with 404 producing a clear exudate and 413 generating a bright yellow exudate on the colony surface (Figure 1), both of which are characteristic exudate colors of *P. chrysogenum*. Microscopically, both produce conidiophores in a typical whorled pattern with branching stipes and terminal phialides harboring conidia formed in chains [39].

**Figure 2:**
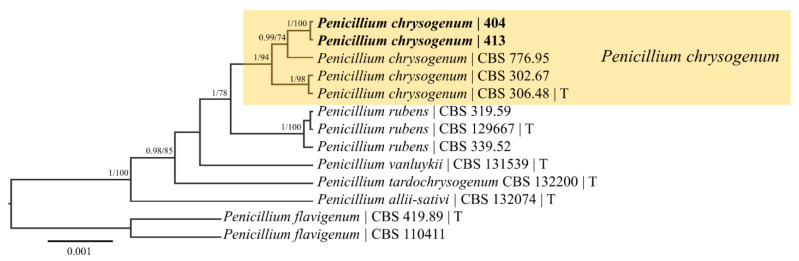
Bayesian maximum clade credibility tree of the concatenated *BenA*, ITS, *rpb1*, *rpb2*, and *CAM* regions of *Penicillium* isolates 404 and 413 and other closely related *Penicillium* taxa. Bayesian posterior probabilities ≥ 90 are displayed followed by bootstrap values greater than 70% for the maximum likelihood (ML) analyses conducted. *Penicillium flavigenum* was chosen as an outgroup taxon based on the analyses by Houbraken et al. [31]. *Penicillium* isolates 404 and 413 formed a monophyletic group with the type specimen of *P. chrysogenum* with high support values. These isolates were genetically separate from all other closely related *Penicillium* species, allowing us to identify the isolates as *P. chrysogenum*. CBS = Fungal Biodiversity Centre; T = sequences were derived from a type specimen. Taxa in bold were sequenced for the current study.

The whole-genome sequencing of 404 and 413 was performed to aid the phylogenetic placement of strains, mine for hygromycin resistance and patulin biosynthetic genes, and add genomic resources for the fungal genetics community to study the unique biology of these two strains. The sequencing reads of 404 and 413 were mapped to the complete, annotated *P. rubens* (previously *P. chrysogenum*) genome P2niaD18 (Table 2) to generate five unitigs for each strain with 89% of raw reads successfully mapped for both. The identification of both isolates was performed using species-specific barcodes *BenA*, ITS, *rpb1*, *rpb2*, and *CaM* to identify each strain (Table 2) [39,40]. Each gene sequence for both 404 and 413 matched *P. chrysogenum* isolates (including numerous type specimens, i.e., DTO235|1, DTO 103E7, etc.) in the NCBI database with 100% query cover and >99% identity. In the phylogenetic analyses, both isolates (404 and 413) formed a monophyletic group with high Bayesian posterior (1) and maximum likelihood bootstrap (94) support with the type specimen of *P. chrysogenum* and other *P. chrysogenum* specimens from the Fungal Biodiversity Centre (CBS) culture collection (Figure 2). These isolates were genetically separate from all other closely related *Penicillium* species, allowing us to accurately identify them as *P. chrysogenum*.

### 3.2. Penicillium chrysogenum Isolates Are Non-Pathogenic in Apple Fruit

**Figure 3:**
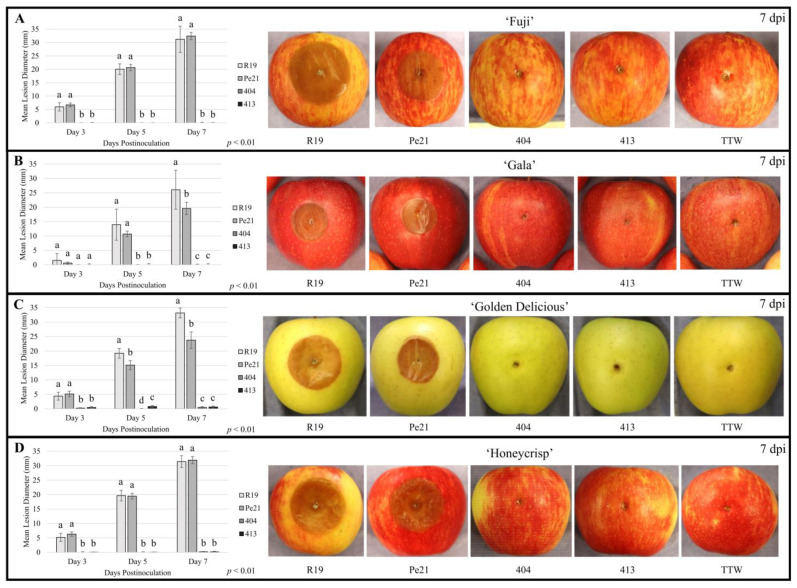
Interaction of *Penicillium* spp. strains R19, Pe21, 404, and 413 with four different apple cultivars. Mean lesion diameters at 3, 5, and 7 days postinoculation (dpi) (left) and representative photos of wounds 7 dpi (right) in (**A**) ‘Fuji’, (**B**) ‘Gala’, (**C**) ‘Golden Delicious’, and (**D**) ‘Honeycrisp’ apple fruits with the mean wound diameter from the tween-treated water (TTW) control subtracted from the mean lesion diameter for each strain. A Kruskal–Wallis test was performed with the Wilcoxon post hoc test (*p* < 0.01, *n* = 10) to determine the significance between isolates at each timepoint, and error bars represent standard deviation. Values with the same letter are not significantly different.

‘Fuji’, ‘Gala’, ‘Golden Delicious’, and ‘Honeycrisp’ apples were wounded and inoculated with R19, Pe21, 404, and 413 strains. Apples inoculated with the *P. expansum* strains showed characteristic brown, watery lesion development radiating out from the inoculation point (Figure 3) [23,27]. The mean lesion diameter for R19 and Pe21 after 3 days was higher than both the 404 and 413 mean diameters for all apple cultivars, which were indistinguishable from the TTW control. By 7 days postinoculation, 404 and 413 maintained no visible lesion or blue mold symptoms beyond the inoculation site (Figure 3). Apple wounds inoculated with each strain were excised and plated on the PDA medium and PDA amended with hygromycin (100 ppm) to determine if the inoculum was viable (Table S1). All samples from apple fruit showed copious fungal growth on the PDA plates, while only the 404 and 413 samples were able to form colonies on the PDA amended with hygromycin. Thus, all the inoculated strains remained viable while in apple fruit, regardless of disease symptomatology.

To determine if 404 and 413 were unique in their non-pathogenic phenotype in apple, we compared them to curated type specimens. Thus, we performed apple inoculation experiments with three *P. rubens* and four *P. chrysogenum* type specimens (Table 1). When inoculated into each apple cultivar, these strains also did not show lesion formation after 7 days of inoculation . Incubation was extended to 14 days to ensure no rot symptoms were observed, which is often the case for slower, weaker pathogens of apple fruit. After 14 days at room temperature, the inoculation sites showed small brown regions that extended radially outward from the point of inoculation, yet the lesion area appeared dry with very limited tissue collapse. This occurred most frequently in ‘Golden Delicious’ apples but inconsistently across isolates in ‘Fuji’ and ‘Honeycrisp’ cultivars (Table S2). After extending the incubation period to 14 days for R19, 404, and 413, the lesions for R19 were roughly the diameter of the entire apple in all cases with extremely liquified maceration of tissue observed routinely with *P. expansum* infection. Both 404 and 413 mimicked the type specimens on symptom development and cultivar susceptibility and were often difficult to differentiate from water-only controls.

### 3.3. Penicillium chrysogenum Isolates Have Comparable Growth Rate to P. expansum In Vitro

To confirm that the lack of symptom development in apple fruit was not a function of poor growth rate, the same conidial suspensions that were used for apple fruit inoculations were also used to inoculate the PDA plates (Figure 1). Then, 14 h postinoculation, all isolates were above 90% germinated, and by 24 h, all were completely germinated. After 7 days of growth on the PDA, R19 had an average colony diameter of 32.2 mm and Pe21 had an average diameter of 49.0 mm, whereas 404 and 413 were an average of 45.5 mm and 45.0 mm in diameter, respectively. Overall, all strains achieved 100% germination by 24 h postinoculation, with 404 and 413 generating colonies of similar size to the *P. expansum* strains after 7 days.

### 3.4. Penicillium chrysogenum Isolates Reduce Penicillium expansum Development in Apple Fruit

**Figure 4:**
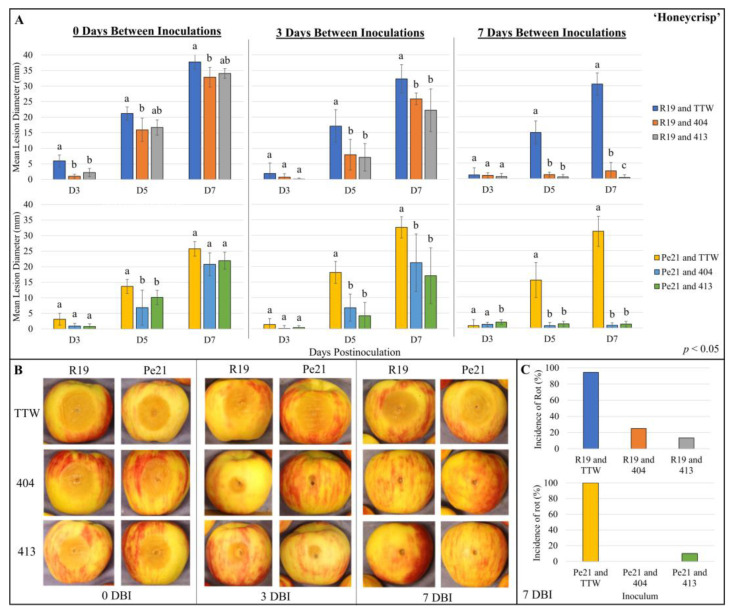
‘Honeycrisp’ apples co-inoculated with *P. chrysogenum* and *P. expansum* isolates at different times: (**A**) Mean lesion diameter of wounds inoculated with *P. expansum* R19 and Pe21 at (left to right) 0, 3, and 7 days after *P. chrysogenum* 404 and 413 inoculation into wounded ‘Honeycrisp’ apples (0, 3, and 7 DBI). All measurements were taken at 3, 5, and 7 days (D3, D5, and D7) postinoculation of the *P. expansum* inoculum, with the mean wound diameter of TTW-inoculated fruit subtracted from each strain set. Data are presented from a representative trial, and error bars represent the standard deviation for each trial (*n* = 5). An ANOVA test with Tukey’s HSD post hoc test (*p* < 0.05) or a Kruskal–Wallis test with Wilcoxon post hoc test (*p* < 0.05) was performed to determine the significance between strains at each timepoint. Values with the same letter are not significantly different. (**B**) Representative photos of lesions formed 7 days after inoculation of R19 (left) or Pe21 (right) into wound 0, 3, and 7 days (0, 3, 7 DBI) after initial inoculation of (top to bottom) TTW control, 404, or 413. (**C**) Percent incidence of rot observed 7 days after *P. expansum* inoculation. The percent incidence shown is from both trials at the 7 DBI timepoint. DBI = days between inoculations, TTW = tween-treated water.

**Figure 5:**
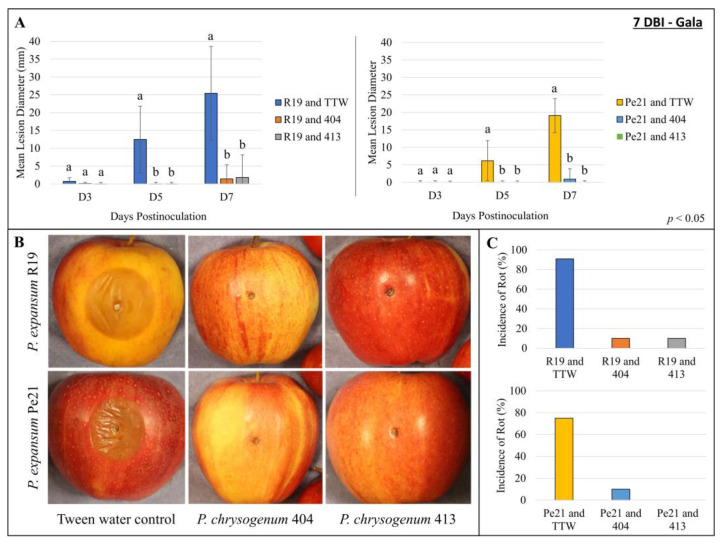
‘Gala’ apples co-inoculated with *P. chrysogenum* and *P. expansum* isolates at different times: (**A**) Mean lesion diameter from wounds inoculated with *P. expansum* R19 and Pe21 at 7 days after *P. chrysogenum* 404 and 413 inoculation into wounded ‘Gala’ apples (7 DBI). All measurements were taken at 3, 5, and 7 days (D3, D5, and D7) postinoculation of the *P. expansum* inoculum, with the mean wound diameter of TTW-only inoculated fruit subtracted from each. Data are presented as the mean lesion diameter from a representative trial (*n* = 5), and error bars represent the standard deviation. A Kruskal–Wallis test was performed with the Wilcoxon post hoc test (*p* < 0.05) to determine the significance between inoculum at each timepoint. Values with the same letter are not significantly different. (**B**) Representative photos of lesions formed 7 days after inoculation of R19 (top) or Pe21 (bottom) into the wound 7 days after the initial inoculation of (left to right) TTW control, 404, or 413. (**C**) Percent incidence of rot observed across both trials 7 days postinoculation of the *P. expansum* isolates (7 DBI). DBI = days between inoculations. TTW = tween-treated water.

To determine whether the avirulent *P. chrysogenum* could inhibit or block the virulent *P. expansum* isolates, the co-inoculation of the two species was performed in ‘Honeycrisp’ apples. The isolates were also co-inoculated with temporal delay to determine the efficacy of *P. chrysogenum* establishment in apple prior to *P. expansum* inoculation. To encourage the establishment of the *P. chrysogenum* isolates, 404 and 413 conidia were added at 100× the concentration of *P. expansum*. The inoculation of *P. expansum* spores was carried out immediately after *P. chrysogenum* (0 days between inoculations; 0 DBI), 3 days after *P. chrysogenum* (3 DBI), or 7 days after *P. chrysogenum* (7 DBI). For the 0-day delay apples, the reduction in *P. expansum* isolates was minimal, with no significance between Pe21 combined with 404 and Pe21 combined with 413 by day 7, and for R19, a variable reduction was observed between trials (Figure 4 and Figure S1). In the 3-day delay, the lesions were more significantly reduced for both R19 and Pe21 when combined with either 404 or 413, yet blue mold lesions were still developed by the 7th day. Finally, with the 7-day delay between *P. chrysogenum* and *P. expansum*, all of the interactions show the most significant reduction and minimal lesion formation by the 7th day. To determine if our observations in ‘Honeycrisp’ held in another popular apple fruit variety, ‘Gala’ apples were used to look for temporal impact on the efficacy of *P. chrysogenum* establishment. Here, the 7-day delay was applied between isolates, as this had the largest reduction in blue mold decay (Figure 5 and Figure S2). Similar to the 7-day delay scenario in ‘Honeycrisp’, the *P. expansum* lesions were significantly reduced with *P. chrysogenum* and showed very minimal lesion formation compared to the controls with just *P. expansum* and TTW.

### 3.5. Penicillium chrysogenum Isolates Do Not Inhibit the Growth of Penicillium expansum In Vitro

**Figure 6:**
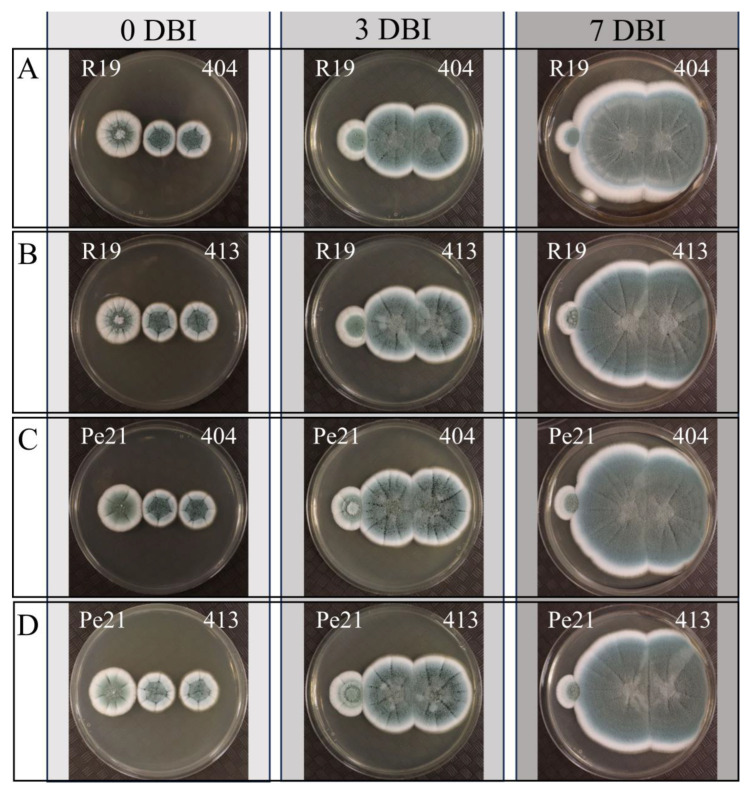
Growth of *P. chrysogenum* and *P. expansum* isolates together on agar medium. Plates inoculated with *P. chrysogenum* isolates 404 and 413 (center and right colonies on each plate) were subsequently inoculated with *P. expansum* isolates R19 and Pe21 (left colony on each plate) 0 (left column), 3 (center column), and 7 (right column) days after inoculation of the *P. chrysogenum* isolates. The combinations shown are (**A**) R19 with 404, (**B**) R19 with 413, (**C**) Pe21 with 404, and (**D**) Pe21 with 413. Colonies were photographed 3 days after adding the *P. expansum* isolates. DBI = days between inoculations.

To further understand the relationship between *P. chrysogenum* and *P. expansum*, each isolate was grown in proximity to the other species on the PDA plates. Isolates 404 and 413 were plated in the center, with the inoculation of *P. expansum* isolates R19 and Pe21 immediately (0 days), after 3 days, or after 7 days of *P. chrysogenum* growth to mimic the timeline of interaction within the apple fruit (Figure 6). Combinations of isolates across all temporal inoculations showed no visible antagonism with either species (e.g., inhibition of growth), and colonies grew into one another by 7 days. 

### 3.6. Penicillium chrysogenum Isolates Are Resistant to Hygromycin and Patulin

**Figure 7:**
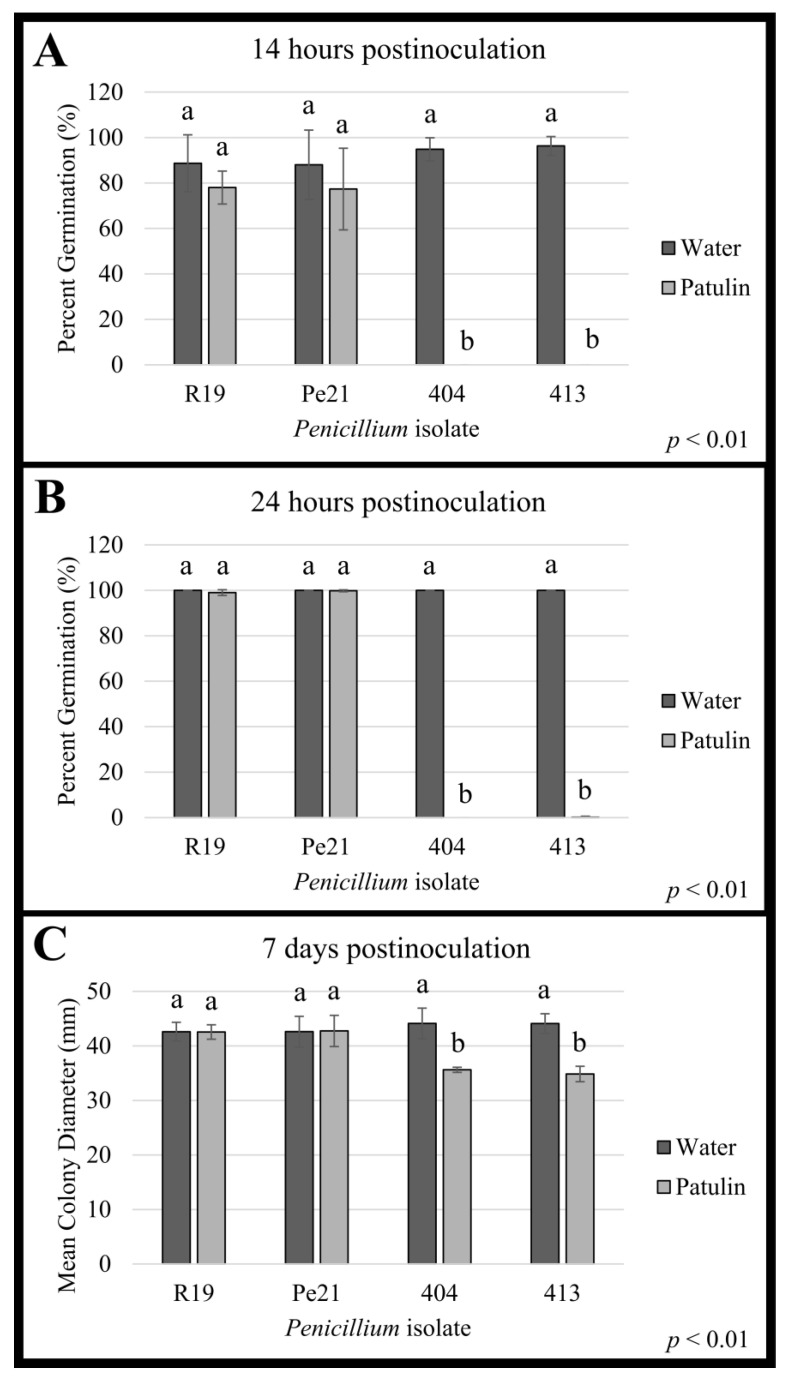
Conidial germination and radial growth of *Penicillium* spp. isolates following exogenous patulin treatment. Germination of *Penicillium expansum* R19 and Pe21, and *P. chrysogenum* 404 and 413 conidia (**A**) 14 h and (**B**) 24 h after exposure to water or patulin (138 µg/spore suspension), and (**C**) radial growth of the four isolates 7 days postinoculation. A Student’s *t*-test or Mann–Whitney U test was performed to determine the significance between treatments for each individual isolate (*p* < 0.01, *n* = 6), and error bars represent standard deviation. Values with the same letter are not significantly different.

Because isolates 404 and 413 were originally isolated from hygromycin-amended media, each isolate was tested for resistance to multiple antimicrobial compounds, including hygromycin, using previously published concentrations as discriminatory doses [14,26,41,42]. The compounds utilized were patulin (PAT), thiabendazole (TBZ), fludioxonil (FLU), pyrimethanil (PYR), difenaconazole (DIF), and hygromycin (HYG). Four isolates were tested against these antimicrobial compounds: 404, 413, Pe21, and R19. After 7 days of growth on the amended medium, both 404 and 413 were able to grow on HYG, while Pe21 and R19 could not (Figure S3; Table S3). Colony growth was visible for all *Penicillium* isolates after PAT exposure after 7 days of growth (Figure 7, Table 1). R19 and Pe21 were observed to germinate completely within the first 24 h after PAT exposure, while both 404 and 413 had significantly delayed germination (Figure 7). Consequently, colony growth for 404 and 413, while detectable, was smaller when initially exposed to patulin than to water, while both R19 and Pe21 had statistically insignificant differences in colony size between treatments. All other antimicrobial compounds inhibited the growth of all the *Penicillium* isolates tested (Table S3), showing that the isolates did not have a multidrug-resistant phenotype.

To understand the basis of the hygromycin resistance in the two *P. chrysogenum* isolates, their genomic data were used to search for the homologs of previously identified hygromycin-resistant genes encoding two aminoglycoside phosphotransferases (APHs), specifically APH(4)-Ia and APH(7″)-Ia [43,44]. However, both genes encoding APH(4)-Ia and APH(7″) were absent in each strain. *P. chrysogenum* NCBI sequences also lack both genes, using both nucleotide and amino acid BLAST search parameters. The closest match in the NCBI database with 23% identity and 49% query cover, which was mirrored in the 404 and 413 sequence data, was to APH(3′), which has not been shown to confer HYG resistance due to structural differences in the protein [43]. Therefore, to determine whether the hygromycin-resistant phenotype was conserved across type specimens within the species of *P. chrysogenum* and *P. rubens*, all seven isolates were grown on hygromycin-amended PDA. After 7 days, all were observed to form colonies and thus were deemed resistant (Table 1).

### 3.7. P. chrysogenum Isolates Do Not Possess an Intact Canonical Patulin Biosynthetic Cluster

**Figure 8:**
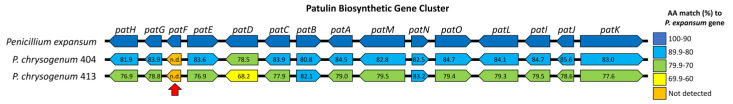
Patulin biosynthetic gene cluster incomplete in *Penicillium chrysogenum* isolates 404 and 413. The amino acid percent similarity of each locus in *P. chrysogenum* 404 and 413 compared to the known canonical *P. expansum* patulin biosynthetic gene cluster. The arrow indicates the genes missing from the two *P. chrysogenum* genomes. N.d. = not detected.

To determine the genomic potential of these strains to produce patulin, a known biosynthetic gene cluster (from *P. expansum* MD-8 [45]) was mined in the genome sequences of R19 (retrieved from NCBI, GenBank Accession: GCA_004302965.1), Pe21 (retrieved from NCBI, genome d1, assembly ASM76973v1), 404, and 413. For patulin, a complete biosynthetic pathway was found for R19 and Pe21, yet only partial clusters were present for isolates 404 and 413 at the nucleotide level. Therefore, each gene from the original cluster was queried in the NCBI BLASTp database against *P. chrysogenum* to find amino acid matches within those available genomes. The top matches were then used to map against 404 and 413 to determine the presence of the gene (Figure 8). Each translated gene within both isolates maintained 60 to 80% amino acid similarity to those within the cluster, except for the gene *patF*, which was unable to be found at the nucleotide or amino acid level.

## 4. Discussion

Blue mold decay is an economically impactful, problematic disease that causes food waste and product loss during pome fruit storage and product processing industries. This is exacerbated by the increased occurrence of fungicide resistance in *Penicillium expansum* to all four labeled postharvest fungicides (thiabendazole, pyrimethanil, fludioxonil, and difenoconazole) [11,12,13,14,15,16]. Alternatives to fungicides have more recently been used in the form of biocontrol agents, such as Bio-Save^®^ [22]. Here, we isolated two *Penicillium* species, designated 404 and 413, with the goal of analyzing them as competitive agents against *P. expansum* infection during apple fruit infection. We determined that both 404 and 413 are *P. chrysogenum* species using whole-genome sequencing and multilocus phylogeny with type specimens. They both are non-pathogenic in apple fruit but grow on the PDA medium comparatively to the well-adapted, highly aggressive *P. expansum* strains. This is important as fungal fitness and growth in axenic culture were not compromised, yet their ability to infect apple fruit was abated, which is reminiscent of an incompatible plant–pathogenic interaction. Other type specimens of both *P. chrysogenum* and the sister species *P. rubens* showed their lack of infection capability. Hence, the potential for these isolates to occupy the same niche and outcompete the wild-type strains of the highly virulent blue mold pathogens (e.g., *Penicillium expansum*) was plausible. Therefore, the two *P. chrysogenum* isolates were inoculated into apples, followed by the *P. expansum* isolates inoculated into the same wound immediately, with a 3-day or 7-day delay after the *P. chrysogenum* isolates. The co-inoculation of the two species resulted in a reduction in the *P. expansum* rot. Furthermore, the establishment of *P. chrysogenum* with the increased delay afforded better protection against blue mold decay, and the phenomenon was observed across both ‘Honeycrisp’ and ‘Gala’ commercial apple cultivars.

There have been some sporadic reports of *P. chrysogenum* causing blue mold decay of apple [46,47], and there have been reports of *P. chrysogenum* in apple storage warehouses at low incidence levels [48,49]. However, the strains shown here produce very small, dry, brown, localized lesions that form >7 days and are atypical compared to infection caused by other well-suited blue mold fungi. Based on the lack of symptom development, it is likely that apple fruits are more capable of combatting *P. chrysogenum*, resulting in much slower and smaller symptom development, reminiscent of an incompatible host–pathogen interaction. A species-specific response to the host could explain the lack of *P. chrysogenum* infection, as has been previously shown in citrus with *P. expansum* and *P. digitatum*. While *P. digitatum* was able to infect citrus in part due to oxidative burst suppression, *P. expansum* was unable to suppress the fruit defense mechanism [50]. Alternatively, it is possible that some previous reports of *P. chrysogenum* infections in apple were instead due to another species within the genus, as the use of multiple genetic markers is necessary for accurate and effective *Penicillium* speciation [51]. Overall, we confirm that their impact is minimal in apple fruit for these *P. chrysogenum* isolates and the type specimens of both *P. chrysogenum* and *P. rubens*. Therefore, this phenomenon provides a unique opportunity to study disparities concerning infection biology in addition to their potential to serve as biocontrol agonists.

The two newly discovered *P. chrysogenum* isolates examined in this study were obtained from hygromycin-amended media in the laboratory. Hygromycin resistance has been attributed to the presence of two aminoglycoside phosphotransferases (APHs), specifically APH(4)-Ia and APH(7″)-Ia [43,44], and is often exploited as a genetic tool for fungal selection using the *Agrobacterium tumefaciens*-mediated transformation or as a dominant selectable marker via protoplast gene transformation studies [52]. Oddly enough, the two APH-encoding genes were not found in either the 404 or 413 genomes. However, spontaneous resistance to hygromycin resistance has been shown in *Monilinia fructicola*, but the mode of action for the resistance was not determined [53]. Even so, the phenotypic challenges of *M. fructicola* isolates (slowed growth, diminished sporulation, reduced melanization, or sensitivity to demethylation fungicides) were not observed in either 404 or 413 [53]. Furthermore, all four type specimens of *P. chrysogenum* and all three *P. rubens* specimens used in this study were also resistant to hygromycin, suggesting a species-level resistance that is conserved. This resistance was also previously observed for a *P. chrysogenum* isolate during the development of tools for genetic mutation in filamentous fungi [41]. In *P. funicolosum*, efflux was the mechanism touted to enhance antimicrobial resistance, including hygromycin, in combination with carbon source availability [54]. Furthermore, tolerance in 404 and 413 was observed for mycotoxin patulin, previously shown to have antifungal properties [26]. However, this effect is modulated in *P. expansum* and *P. crustosum* via efflux pump activity [26]. Given this, we hypothesize the resistance mechanism for these species may be due to an increase in the efflux of both patulin and hygromycin. This has previously been shown in a variety of organisms, including *P. expansum* [11,13,26,54,55], and is often seen in cases of multidrug-resistant phenotypes concerning fungal plant, animal, and human pathogens [56,57]. However, other mechanisms of resistance are also possible and likely (i.e., metabolism, sequestration, and binding affinity), as outlined by Hu and Chen [55], showing further need for investigation.

There have been some reports in the literature on the ability of *P. chrysogenum* to produce patulin [58,59,60]. However, it is important to note that each of these reports was based solely upon strains that were identified via morphology and lacked multigene phylogeny or whole-genome data. More recent works show a lack of patulin production by *P. chrysogenum* [47,61]. For *P. chrysogenum*, previous whole-genome sequence comparisons have found variability in secondary metabolite clusters and copy numbers [62]. In this study, the two *P. chrysogenum* strains (404 and 413) did not have an intact patulin biosynthesis gene cluster. This was corroborated by previous findings on the fungus, with *P. chrysogenum* species, including the Wisconsin 54-1255 type specimen, containing low homology or partial clusters for the patulin biosynthetic gene cluster [61,63,64]. Additionally, the limited lesion formation in the apple further supports the lack of patulin production, since a previous study showed patulin alone is responsible for tissue death and contributes toward measurable lesions on apple fruit tissue [26]. These findings show the importance of modern speciation genetic-/genomic-based techniques but may also show variability within the species concerning mycotoxin production. Regardless, the findings here are especially important as biocontrol strain selection would likely require the exclusion of those that could produce harmful toxins like patulin.

The proposed use of *P. chrysogenum* 404 and 413 as antagonists would enable competition against blue mold in the postharvest space with negligible harm to the fruit or contamination of fruit products with patulin. Previous work has investigated *P. chrysogenum* as a biocontrol in storage against *Penicillium*-derived mycotoxins in dried meat products, including a reduction in ochratoxin production by *P. nordicum* in ham [65] and cyclopiazonic acid production by *P. griseofulvum* in sausage [66]. *P. chrysogenum* application also maintained sensory quality and native microbial presence on dried sausage [67]. An antifungal protein (AFP) derived from a *P. chrysogenum* isolate has antimicrobial activity against *Penicillium* and *Aspergillus* in dry-cured meat [68]. Finally, a study by Delgado and colleagues used *P. chrysogenum* spores on apple and showed effectiveness against *P. expansum* in ‘Royal Gala’ cultivars [69]. Determining the suitability of these strains for biocontrol use is one major purpose of these works; however, further pilot testing via competition on additional commercial apple cultivars and other pome fruit, application to apple bins and other surfaces in the fruit postharvest system, and testing under storage conditions with a controlled atmosphere would need to be performed to determine if these isolates are viable candidates for biocontrol. Even so, the tolerance to antimicrobials produced by other microbes, including the patulin and hygromycin presented here, could improve fitness in this competitive dynamic. Whether *P. chrysogenum* could also compete with and prevent the infection of other postharvest pathogens in apple that cause rot and produce mycotoxins is unknown, but it could expand the utility of these fungi further. Ultimately, the findings from the apple virulence assay in conjunction with the antimicrobial tolerance shed new light on expanding the utility of these organisms and their applications in the postharvest industry.

Current biocontrol products in the industry have multiple effective modes of action, including direct antagonism, the stimulation of host defense, secondary metabolite production, and niche competition. For the 404 and 413 isolates, we provide evidence suggesting that their efficacy is most likely due to the establishment of *P. chrysogenum* that allows for niche colonization and nutrient uptake monopoly within the wound of the fruit. This was demonstrated through the temporal application of the fungi, where a longer time allowed for the increased establishment of *P. chrysogenum*. Additionally, the plate competition assay did not show any inhibition zone that would indicate direct antagonism or the production of antifungal compounds against *P. expansum*. However, further investigation into the metabolites generated during this interaction could yield some potential antagonistic compounds that provide a competitive advantage for *P. chrysogenum*. It is also important to note that the ratio of fungal inoculum added was 100 times higher in the *P. chrysogenum* application than in the subsequent *P. expansum* application to ensure the thorough coverage of the wound, as previous work has effectively demonstrated [69]. In an apple storage facility, the initial inoculum of *P. expansum* spores will likely be further reduced from our artificial inoculation, which could allow for further effectiveness of the *P. chrysogenum* application. Furthermore, apple maturity has been shown to impact infection and mycotoxin production, with later-stage apples having increased susceptibility due to high sugar content, low tissue firmness, and other factors [70]. Apples harvested, treated, and stored at the beginning of the season are far less mature than those used in this study, and would likely show further improvement with *P. chrysogenum* application than those used here. Finally, the temperatures used in this study were for an accelerated study of the fungal interplay on the apple. However, in a storage facility with a 1–4 °C atmosphere, the *P. chrysogenum* application may have improved efficacy beyond the significant reduction in blue mold shown here, as *P. expansum* will have a heavy growth penalty in those cold conditions. We therefore suggest that *P. chrysogenum* is a promising alternative to assist in the mitigation of blue mold decay during postharvest apple fruit storage, given the practical testing of these strains into the latest industry production practices.

## 5. Conclusions

Blue mold is a major problem for the postharvest pome fruit storage, packing, and processing industries. Current mitigation strategies are not sufficient for the elimination of the disease or the abatement of patulin production and accumulation in processed fruit products. Biological control organisms are effective alternatives to conventional fungicides used and are especially of interest for organic producers and to combat infection with fungicide-resistant isolates. The two *P. chrysogenum* isolates in this study have promising potential to be optimized as biocontrols against blue mold decay due to their ability to outcompete *P. expansum* in vivo and colonize the postharvest apple niche without causing rot symptoms or producing the mycotoxin patulin.

## Figures and Tables

**Table 1:** Fungal isolates used in this study.

Fungal Isolate	Patulin	Hygromycin	Pathogenicity in Apple Fruit
*Penicillium expansum* R19	Resistant	Susceptible	Yes
*Penicillium expansum* Pe21	Resistant	Susceptible	Yes
*Penicillium chrysogenum* isolate 404	Resistant	Resistant	No
*Penicillium chrysogenum* isolate 413	Resistant	Resistant	No
*Penicillium chrysogenum* DTO 275-D7	Not tested	Resistant	No
*Penicillium rubens* DTO 346-D4	Not tested	Resistant	No
*Penicillium chrysogenum* DTO 382-H5	Not tested	Resistant	No
*Penicillium rubens* DTO 397-I3	Not tested	Resistant	No
*Penicillium chrysogenum* DTO 402-A9	Not tested	Resistant	No
*Penicillium rubens* DTO 405-A6	Not tested	Resistant	No
*Penicillium chrysogenum* DTO 431-E3	Not tested	Resistant	No

**Table 2:** Draft whole-genome sequencing, assembly, and identification of *Penicillium* 404 and 413 isolates using short-read Illumina-based technology.

Sequenced Isolate	Raw Reads	% GC	Reads Mapped to Genome Reference ^a^	Barcode Loci ^b^	Identification
*Penicillium* isolate 404	14,127,414	48.0	12,628,224	*BenA*, ITS, *rpb1*, *rpb2*, *CaM*	*P. chrysogenum*
*Penicillium* isolate 413	17,801,280	47.9	15,883,217	*BenA*, ITS, *rpb1*, *rpb2*, *CaM*	*P. chrysogenum*

^a^ Reference sequence = *P. rubens* P2niaD18. ^b^ Species were confirmed using species-specific barcodes, and each locus exhibited a minimum 99% identity and 100% query cover match to *P. chrysogenum.*

## Data Availability

The whole-genome sequencing data generated in this study are openly available in the NCBI Sequence Read Archive with the BioProject accession number PRJNA945086, or at https://www.ncbi.nlm.nih.gov/bioproject/PRJNA945086. All other generated data are contained within the article and Supplementary Materials.

## Supplementary Materials

The following supporting information can be downloaded at: https://www.mdpi.com/article/10.3390/microorganisms11112792/s1, Figure S1: ‘Honeycrisp’ apples co-inoculated with *P. chrysogenum* and *P. expansum* isolates at different times; Figure S2: ‘Gala’ apples co-inoculated with *P. chrysogenum* and *P. expansum* isolates at different times; Figure S3: Conidial germination and radial growth of *Penicillium* spp. isolates following hygromycin treatment; Table S1: Re-isolation and growth of *Penicillium* spp. strains on selective media, containing hygromycin B, recovered from apple fruit cultivars ‘Fuji’, ‘Gala’, ‘Golden Delicious’, and ‘Honeycrisp’ 7 days postinoculation; Table S2: Wound sizes (expressed as mean lesion diameter in mm) caused by *P. chrysogenum* and *P. rubens* spore suspensions inoculated in wounded apple fruit 14 days postinoculation; Table S3: Growth of *Penicillium* spp. strains on selective media, containing different antimicrobial compounds at specific discriminatory doses, after 7 days.
