# Supplementary material for: Avirulent Isolates of Penicillium chrysogenum to Control the Blue Mold of Apple Caused by P. expansum
Source: Microorganisms. 2023 Nov 17;11(11):2792. doi: 10.3390/microorganisms11112792 (PMC10673114; doi:10.3390/microorganisms11112792)
Supplement: Supplementary file 1 [file microorganisms-11-02792-s001.zip › microorganisms-2649430-supplementary.pdf]

Supplementary Materials

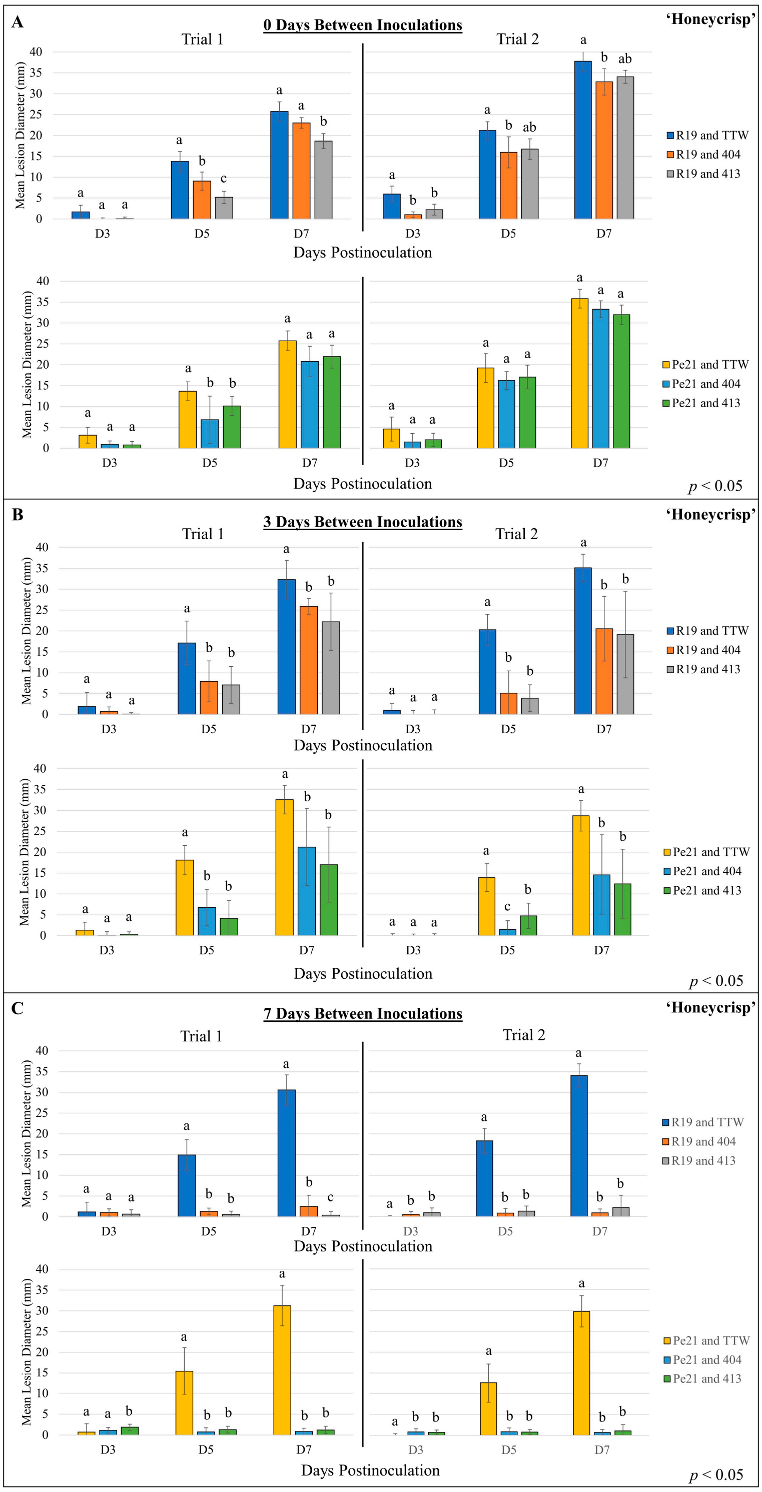

Figure S1. 'Honeycrisp' apples co-inoculated with *P. chrysogenum* and *P. expansum* isolates at different times. Mean lesion diameter of wounds inoculated with *P. expansum* R19 and Pe21 at A) 0, B) 3, and C) 7 days after *P. chrysogenum* 404 and 413 inoculation into wounded 'Honeycrisp' apples (0, 3, and 7 DBI). All measurements were collected at 3, 5, and 7 days (D3, D5, and D7) postinoculation of the *P. expansum* inoculum, with the mean wound diameter of TTW-inoculated fruit subtracted from each strain. Error bars represent standard deviation. An ANOVA with Tukey's HSD post hoc test ($p < 0.05$), or a Kruskal–Wallis test with Wilcoxon post hoc test ($p < 0.05$, $n = 5$) was performed to determine significance between inoculum at each timepoint. Values with the same letter are not significantly different. DBI = days between inoculations.

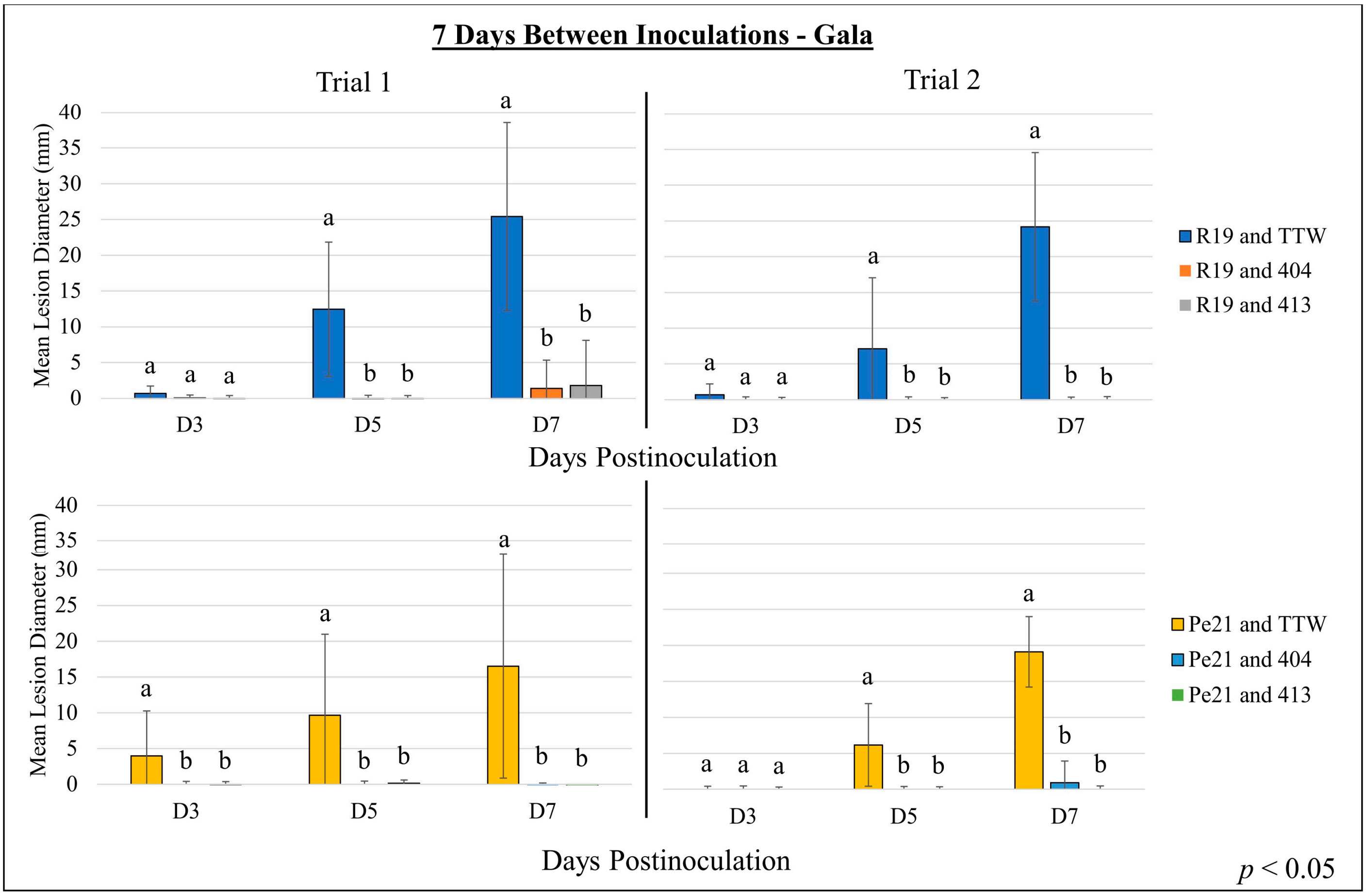

Figure S2. 'Gala' apples co-inoculated with *P. chrysogenum* and *P. expansum* isolates at different times. Mean lesion diameter from wounds inoculated with *P. expansum* R19 and Pe21 at 7 days after *P. chrysogenum* 404 and 413 inoculation into wounded 'Gala' apples (7 DBI). All measurements collected at 3, 5, and 7 days (D3, D5, and D7) postinoculation of the *P. expansum* inoculum, with the mean wound diameter of TTW-only inoculated fruit subtracted from each strain. Error bars represent the standard deviation. A Kruskal–Wallis test was performed with Wilcoxon post hoc test ($p < 0.05$, $n = 5$) to determine significance between strains at each timepoint. Values with the same letter are not significantly different. DBI = days between inoculations.

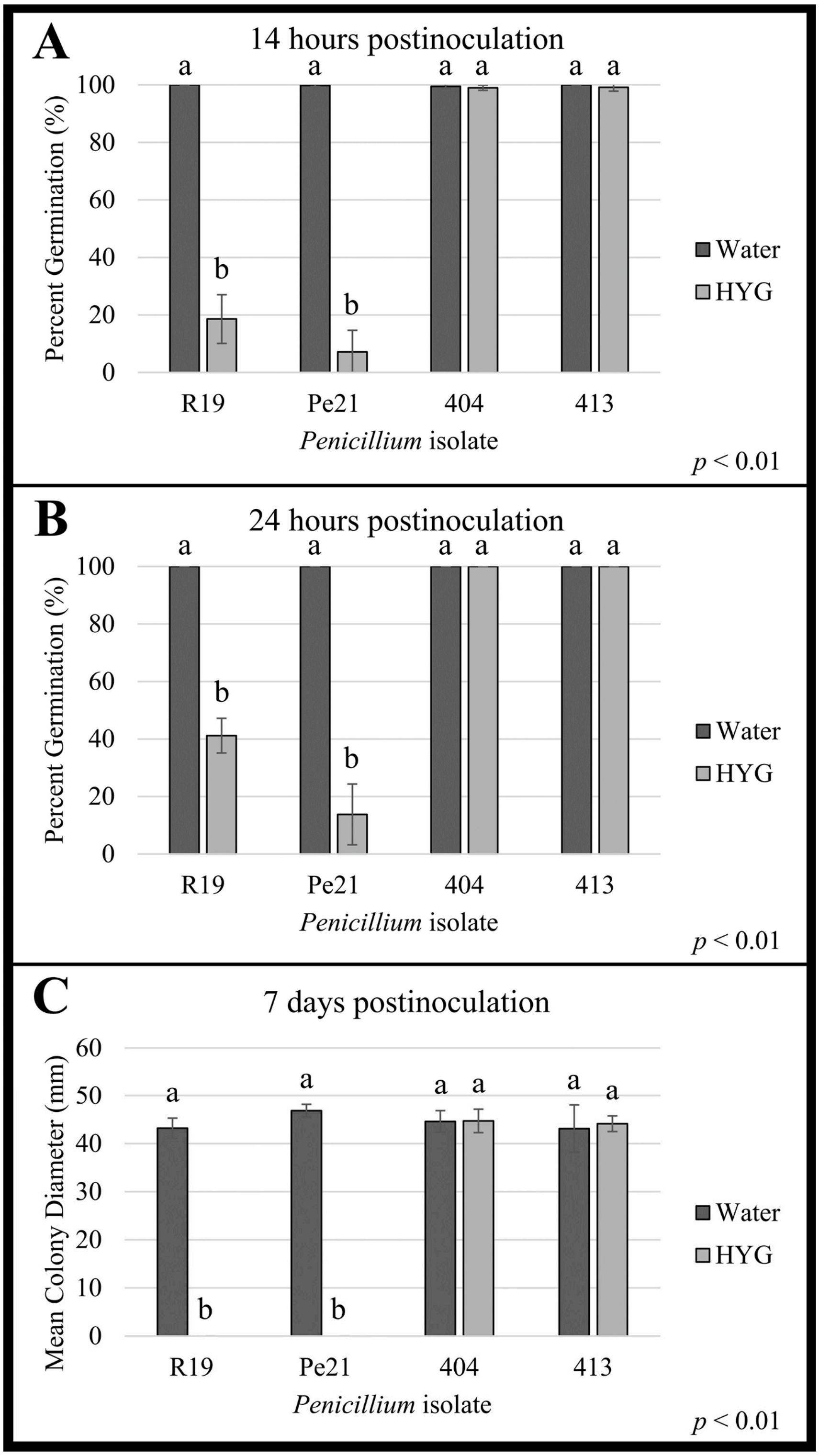

Figure S3. Conidial germination and radial growth of *Penicillium* spp. isolates following hygromycin treatment. Germination of *Penicillium expansum* R19 and Pe21, and *P. chrysogenum* 404 and 413 after A) 14 hours and B) 24 hours after exposure to water or hygromycin (HYG; 100 ppm), and C) Radial growth of the four fungal isolates 7 days postinoculation. A Student's *t*-test or Mann–Whitney U test was performed to determine significance between treatments for each individual isolate ($p < 0.01$, $n = 6$), and error bars represent standard deviation. Values with the same letter are not significantly different.

Table S1. Re-isolation and growth of *Penicillium* spp. strains on selective media, containing hygromycin B, recovered from apple fruit cultivars 'Fuji', 'Gala', 'Golden Delicious', and 'Honeycrisp' 7 days postinoculation[a]

Strain	Apple Cultivar	PDA	PDA + hygromycin B (100 ppm)
Penicillium expansum R19	'Fuji'	+	-
	'Gala'	+	-
	'Golden Delicious'	+	-
	'Honeycrisp'	+	-
Penicillium expansum Pe21	'Fuji'	+	-
	'Gala'	+	-
	'Golden Delicious'	+	-
	'Honeycrisp'	+	-
Penicillium chrysogenum 404	'Fuji'	+	+
	'Gala'	+	+
	'Golden Delicious'	+	+
	'Honeycrisp'	+	+
Penicillium chrysogenum 413	'Fuji'	+	+
	'Gala'	+	+
	'Golden Delicious'	+	+
	'Honeycrisp'	+	+
TTW	'Fuji'	-	-
	'Gala'	-	-
	'Golden Delicious'	-	-
	'Honeycrisp'	-	-

[a]Growth on agar medium indicated with '+', no growth indicated with '-'. TTW = Tween-Treated Water control. PDA = Potato Dextrose Agar.

Table S2. Wound sizes (expressed as mean lesion diameter in mm) caused by *P. chrysogenum* and *P. rubens* spore suspensions inoculated in wounded apple fruit 14 days postinoculation.

Isolate	'Golden Delicious'	'Honeycrisp'	'Fuji'
P. chrysogenum DTO 275-D7	3.5 ± 0.72	0	0.76 ± 0.44
P. chrysogenum DTO 431-E3	3.34 ± 1.51	0	0
P. chrysogenum DTO 402-A9	3.82 ± 1.29	0	0
P. chrysogenum DTO 382-H5	3.4 ± 1.10	0 ± 1.97	0
P. rubens DTO 405-A6	3.7 ± 0.85	0	0
P. rubens DTO 397-13	3.7 ± 0.63	0	0
P. rubens DTO 346-D4	5.82 ± 0.33	0.48 ± 5.46	0.72 ± 4.34
P. chrysogenum 404	2.82 ± 1.63	0	0.692 ± 0.42
P. chrysogenum 413	2.54 ± 1.50	0.98 ± 0.71	1.24 ± 0.28
P. expansum R19	52.02 ± 8.87	61.3 ± 2.92	54.76 ± 3.27

[a]Type-specimens of *P. chrysogenum* and *P. rubens* were inoculated into apple fruits and compared to isolates 404, 413, and *P. expansum* R19. Lesion diameters were recorded 14 days postinoculation. Table provides representative data for one trial. All data represented with subtracted TTW-inoculated control wound. Standard deviation presented after "±".

Table S3. Growth of *Penicillium* spp. strains on selective media, containing different antimicrobial compounds at specific discriminatory doses, after 7 days[a]

**Fungicide (medium)**	***Penicillium* spp. isolate**			
	**R19**	**Pe21**	**404**	**413**
**TBZ (PDA)**	-	-	-	-
**FLU (PDA)**	-	-	-	-
**PYR (SA)**	-	-	-	-
**DIF (MEA)**	-	-	-	-
**HYG (PDA)**	-	-	+	+
**PAT (PDA)**	+	+	+	+
**PDA control**	+	+	+	+
**SA control**	+	+	+	+
**MEA control**	+	+	+	+

[a]Growth on agar indicated with '+', no growth indicated with '-'. PDA = Potato Dextrose Agar; SA = Sugar Agar; MEA = Malt Extract Agar; TBZ = thiabendazole (10 ppm); PYR = pyrimethanil (0.5 ppm); DIF = difenoconazole (2.5 ppm); FLU = fludioxonil (0.5 ppm); HYG = hygromycin (100 ppm)
